# Diesel-born organosulfur compounds stimulate community re-structuring in a diesel-biodesulfurizing consortium

**DOI:** 10.1016/j.btre.2020.e00572

**Published:** 2020-11-23

**Authors:** Maysoon Awadh, Huda Mahmoud, Raeid M.M. Abed, Ashraf M. El Nayal, Nasser Abotalib, Wael Ismail

**Affiliations:** a Environmental Biotechnology Program, Life Sciences Department, College of Graduate Studies, Arabian Gulf University, Manama, Bahrain; b Department of Biological Sciences, Faculty of Science, Kuwait University, Kuwait; c Biology Department, College of Science, Sultan Qaboos University, Muscat, Oman

**Keywords:** Biodesulfurization, Dibenzothiophene, *Rhodococcus*, Diesel, 4S pathway, Microbial consortia

## Abstract

We enriched and characterized a biodesulfurizing consortium (designated as MG1). The MG1 consortium reduced the total sulfur of diesel by 25 % and utilized each of the diesel-born compounds dibenzothiophene (DBT), benzothiophene (BT), 4-methyldibenzothiophene (4-MDBT) and 4, 6-dimethyldibenzothiophene (4, 6-DMDBT) as a sole sulfur source. MiSeq analysis revealed compositional shifts in the MG1 community according to the type of the sulfur source. A DBT-grown MG1 culture had *Klebsiella*, *Pseudomonas*, *Rhodococcus* and *Sphingomonas* as the most abundant genera. When diesel or 4, 6-DMDBT was provided as a sole sulfur source, *Klebsiella* and *Pseudomonas* spp. were the most abundant. In the BT culture, *Rhodococcus* spp. were the key biodesulfurizers, while *Klebsiella*, *Pseudomonas* and *Sphingomonas* spp. dominated the 4-MDBT-grown consortium. MG1 also utilized 2–hydroxybiphenyl (the product of the 4S biodesulfurization pathway) where *Pseudomonas* spp. uniquely dominated the consortium. The data improves our understanding of the sulfur source-driven structural adaptability of biodesulfurizing consortia.

## Highlights

• A biodesulfurizing consortium consisting of biodesulfurizers and non-biodesulfurizers.
• Sulfur source-driven compositional shifts in a biodesulfurizing consortium.
• A biodesulfurizing consortium harbors a non-destructive desulfurization pathway.

## 1 Introduction

The production of transportation fuels with near–zero–sulfur content has become mandatory due to the hazardous impact of sulfur oxide emissions resulting from fuels combustion on the environment and public health [1,2]. Oil refineries commonly implement hydrodesulfurization to remove sulfur from petroleum-derived fuels. However, this process is associated with many economical, technical and environmental shortcomings. Among the major drawbacks of hydrodesulfurization are the high cost, the insufficient efficiency towards the most abundant organosulfur compounds in diesel (dibenzothiophene, DBT, and alkylated derivatives), the generation of hazardous spent catalysts and greenhouse gases and the severe treatment conditions [3,4]. Hence, biodesulfurization of fossil fuels has emerged as a green and cost-effective approach to exploit the ability of dedicated microbes to selectively remove sulfur from diesel and other fossil fuels [5,6]. Many biodesulfurization-competent bacteria have been isolated including members of the genera *Rhodococcus*, *Mycobacterium*, *Gordonia*, *Klebsiella*, *Chelatococcus*, *Enterobacter*, and *Sphingomonas*. These bacteria are capable of utilizing one or more of the diesel-born thiophenic compounds, such as DBT, benzothiophene (BT), and their alkylated derivatives, as a sole sulfur source, via the most common biodesulfurization pathway, the 4S pathway (Supplementary Fig. S1) [1,[6], [7], [8]] This pathway proceeds via sequential transformation of DBT to DBT-sulfoxide, DBT-sulfone, 2-hydroxybiphenyl-2′-sulfinate and eventually produces 2-hydroxybiphenyl (2-HBP) as a dead-end product, releasing the sulfur atom of the thiophene ring as sulfite for assimilation [7,[9], [10], [11]]. In a few biodesulfurizing bacteria, 2-HBP is transformed to biphenyl or 2-methoxybiphenyl [8,12,13]. The 4S pathway was initially reported in *Rhodococcus* strain IGTS8 [10,11], which was recently reclassified as *Rhodococcus qingshengii* IGTS8 [14].

Despite more than 30 years of research and development, biodesulfurization has not been applied at an industrial scale yet mainly due to the low catalytic efficiency of the available biodesulfurizing bacteria [4,[14], [15], [16]]. Most of the previous research has focused on axenic cultures of biodesulfurizing bacteria [[17], [18], [19]]. On the contrary, much less efforts have been invested to exploit the advantageous lifestyle of bacterial consortia that could potentially promote the biodesulfurization activity through various microbial interactions [20,21]. In an early study, it was reported that the biodesulfurization activity of a consortium consisting of the biodesulfurizing *Rhodococcus rhodochrous* IGTS8 (later identified as *R. erythropolis* IGTS8) and the non-biodesulfurizing *Enterobacter cloacae* was higher than that of the biodesulfurizing strain IGTS8 alone [22]. Recently, a biodesulfurizing mixed culture was enriched from a hydrocarbon–polluted soil and consisted mainly of species belonging to *Sphingomonas*, *Klebsiella*, *Mycobacterium*, *Rhodococcus*, *Stenotrophomonas*, *Sphingobacterium*, *Pseudomonas*, and *Arthrobacter* [17]. A commercial bacterial consortium consisting of *Alcaligenes faecalis*, *Acinetobacter* sp., *Bacillus* sp., *Komagataeibacter hansenii*, *Oceanobacillus iheyensis*, *Ochrobactrum anthropic*, *Paenibacillus lautus* and *Providencia rettgeri* was used for the biodesulfurization of heavy gas oil and achieved 71.8 % biodesulfurization rate after 6 h of treatment [23]. Although those earlier studies showed the biodesulfurization potential of mixed cultures, they raised the following questions: Which biodesulfurization pathway is adopted by consortia? Do consortia change their composition when exposed to different sulfur sources? How does the biodesulfurization activity of consortia compare with that of model axenic strains? Can consortia desulfurize diesel? Do bacterial consortia affect the hydrocarbon components of the fuel? What is the biodesulfurization substrate range of bacterial consortia? Is it possible to eliminate the inhibitory effect of 2-HBP by using desulfurizing consortia?

In this study, we aimed to answer a couple of those questions. We enriched a biodesulfurization–competent bacterial consortium from seawater in a local lagoon. Using MiSeq amplicon sequencing, we revealed the bacterial composition of this consortium and shifts in its community structure at different phases of growth on various sulfur and carbon sources. We also investigated the biodesulfurization substrate spectrum and elucidated the biodesulfurization pathway. Eventually, we compared the biodesulfurization activity of the consortium to that of the biodesulfurization model strain *Rhodococcus qingshengii* IGTS8 using hydrodesulfurized diesel as a sole sulfur source.

## 2 Materials and methods

### 2.1 Sample collection and media preparation

To enrich biodesulfurizing cultures, water samples from Galali lagoon–Bahrain were collected in April 2014 and used as an inoculum (Supplementary Fig. S2). The lagoon salinity and pH at the time of sampling were 59 ppt (part per trillion) and 7.4, respectively. Sulfur–free chemically defined medium (CDM) was prepared (Supplementary Table S1). The carbon source of the medium was either glucose (10 mM), ethanol (0.1 % v/v), or 2–HBP (0.1 mM), whereas the sulfur source was either MgSO_4_.7H_2_O (1 mM) as an inorganic source, hydrodesulfurized diesel, or any of six other organosulfur compounds (0.1 mM, prepared in ethanol or acetone) including DBT, BT, 4, 6–dimethyldibenzothiophene (4,6–DMDBT), 4–methyldibenzothiophene (4–MDBT), dibenzothiophene sulfone (DBT–sulfone) and dibenzylsulfide (DBS) (Sigma Aldrich–USA, Fluka–Switzerland and Acros–USA). MgCl_2_.6H_2_O (1 mM) was used as a magnesium source when the organosulfur compounds were added either as the sole sulfur and carbon source or as the sole sulfur source.

### 2.2 Enrichment of a biodesulfurizing consortium

Enrichment of biodesulfurizing bacteria was carried out by inoculating water samples (10 mL) into 100 mL of sterilized CDM (in 250–mL Erlenmeyer flasks) supplemented with 0.1 mM DBT (as a sole sulfur source) and 10 mM glucose and (as a carbon source). The enrichment cultures were incubated in an orbital shaker for 7 days, and 1 mL from those initial enrichments was transferred to fresh CDM with the same composition four subsequent times. After the fourth subculture, the enriched culture (designated as MG1) was grown for 24 h (OD_600_ = 0.85–0.9) in CDM–DBT–glucose and samples therefrom were stored in 25 % glycerol stocks at ­– 80°C. To prepare the inoculum for subsequent experiments, the MG1 consortium was grown in CDM (with glucose and DBT) for 24 h, and the cells were harvested, washed, and resuspended in K-phosphate buffer (0.1 M, pH 7).

### 2.3 Growth and substrate range

The ability of the MG1 consortium to grow on and utilize different organosulfur compounds was tested in three sets of cultures. The first set included 10 mM glucose as a carbon source and either DBT, 4–MDBT, BT, DBS, 4, 6–DMDBT, DBT–sulfone, a mixture of organosulfur substrates (DBT, 4–MDBT, BT and 4, 6–DMDBT) or 10 % (vol/vol) of hydrodesulfurized diesel as a sole sulfur source (Table 1). The hydrodesulfurized diesel was provided by Bahrain Petroleum Company (BAPCO). The second set contained each organosulfur compound (0.1 mM) as the sole carbon and sulfur source (without adding any exogenous carbon source). In the third set, the MG1 consortium was grown in CDM containing one of the organosulfur compounds (0.1 mM) (added as solid, no solvent) without adding any exogenous carbon source. The MG1 consortium was also grown in CDM containing either ethanol (0.1 %, vol/vol) or 2–HBP (0.1 mM) as a carbon source and MgSO_4_ (1 mM) as a sole sulfur source. Growth was monitored by measuring culture turbidity (OD_600_) or biomass as gram dry cell weight per litre (g dcw/L). To check for the utilization of the different organosulfur compounds, aliquots of the MG1 cultures (500 μL) were extracted with one volume of ethylacetate. After evaporation of the organic phase, the residue was resuspended in 100 μL of ethanol and analyzed by high performance liquid chromatography (HPLC. Thermo–Dionex–UHPLC–3000) using Acclaim™ C18 column (4.6 × 150 mm, 120 Å, 5 μm particle size). The mobile phase was a mixture of acetonitrile and water (60 % and 80 %) pumped at a flow rate of 1 mL/min. Detection of the substrates and biodesulfurization products was performed with a photodiode array detector (Thermo Scientific, USA) at various wave lengths (233, 235, 248 nm) and retention times were matched using authentic organosulfur substrates and 2–HBP.

**Table 1:** MG1 consortium cultures for community compositional shifts analysis by Illumina–MiSeq amplicon sequencing.

Carbon source	Sulfur source
Glucose (10 mM)	organosulfur compound (0.1 mM) (DBT, 4–MDBT, BT or 4,6–DMDBT)
Glucose	Mixed organosulfur compounds (DBT + 4–MDBT + BT + 4,6–DMDBT)
Glucose	Diesel (10%, vol/vol)
Glucose	MgSO_4_ (1 mM)
Glucose	MgSO_4_ (1 mM) + DBT (0.1 mM)
2–HBP (0.1 mM)	MgSO_4_ (1 mM)
Ethanol (1%)	MgSO_4_ (1 mM)

### 2.4 Bacterial composition of the MG1 consortium

Illumina–MiSeq amplicon sequencing was used to investigate the composition of the MG1 consortium and the temporal (i.e. at different growth phases) shifts in its community structure in the presence of different sulfur and carbon sources as listed in Table 1. The inocula (1%) were prepared from a preculture with 10 mM glucose as a carbon source and 0.1 mM DBT as a sulfur source. All cultures were grown in 250 mL Erlenmeyer flasks containing 100 mL of CDM. Cells were harvested at different growth phases (early-, mid-, late-log and stationary phase) from 10 mL of the cultures by centrifugation at 10,000 rpm for 10 min. The cell pellets were washed once with 0.1 M K–phosphate buffer (pH 7) and resuspended in 0.5 mL nuclease-free water. Genomic DNA was isolated from cell pellets using PowerSoil DNA isolation kit (MOBIO Laboratories, USA) following the manufacturer’s instructions. The DNA extracts were sent to SEQme Company (Dobris, Czech Republic) for Illumina–MiSeq analysis.

Amplicons covering the sequences between positions 515–806 of the 16S rRNA gene for bacteria [24] were generated by PCR using the primers Bac_515 F (GTGYCAGCMGCCGCGGTAA) and Bac_806R (GGACTACNVGGGTWTCTAAT) containing 10 bp barcode sequences at the 5′ ends. Amplicon libraries were generated using the NEBNext® Ultra™ DNA Library Prep kit for Illumina (New England Biolabs, Ipswich, MA) according to the manufacturer’s instructions. The final library quality control was performed on Agilent Bioanalyzer 2100 using High Sensitivity DNA kit. The library was quantified by qPCR using the KAPA Library Quantification kit (Kapa Biosystems, Wilmington, MA) and sequenced on Illumina–MiSeq (Illumina Hayward, CA) for 251 cycles from each end of the fragments (paired-end sequencing) using a MiSeq Reagent kit version 2,500 cycles (Illumina).

Sequence data were demultiplexed and adapters and primers were removed using the software FASTqProcessor version 1.1.4.19846 (http://www.mrdnafreesoftware.com/). Further sequence processing steps were conducted in R version 3.5.2 [25] using the package *dada2* version 1.10.1 [26]. Briefly, sequences were quality filtered at a maximum expected error rate of 3 after trimming both forward and reverse reads to 230 bp (bacterial data set). Error learning, dereplication, and denoising were conducted with default parameters. Forward and reverse reads were merged with a minimum overlap of 10 bp. For chimera detection and taxonomic classification, default settings were selected. Bacterial sequences were classified using the SILVA reference database version 132 [27]. Only non-singleton and non-doubleton sequence variants, hereafter referred to as operational taxonomic units (OTUs), that were classified on phylum level were retained for further analysis. The 16S rRNA gene amplicon sequences are available at the Sequence Read Archive (SRA) under accession number PRJNA631048.

### 2.5 Elucidation of the DBT biodesulfurization pathway

To elucidate the DBT biodesulfurization pathway, DNA from the MG1 consortium was used to detect the 4S pathway genes *(dszAbC)* using gene specific primers [17,28,29]. For the detection of the biodesulfurization intermediates, the MG1 consortium was grown in CDM with glucose (10 mM) and DBT (0.1 mM) as described earlier (in triplicates) and samples (25 mL) were withdrawn at the beginning of the experiment and then after 24, 48, and 72 h. The culture samples were centrifuged (10,000 rpm, 10 min, 4 °C) to remove cells and the cell–free supernatants were extracted with one volume of GC-grade ethyl acetate. The organic phase was evaporated in a rotary evaporator and the residues were suspended in 2 mL of ethyl acetate. For comparison, cultures of the reference strain IGTS8 were prepared and treated in the same way. Ethyl acetate extracts from the MG1 and IGTS8 cultures were analyzed by GC–MS using a Shimadzu gas chromatography system (GC 2010 plus) coupled with a Shimadzu MS–QP2020 mass detector using a Rxi–5 ms column (30 m, 0.25 mm id, 0.4 μm df, Restek, USA). The oven temperature program was set to 1 min at 50 °C with increments of 10 °C/min up to 280 °C, where the oven was held at this temperature for 10 min. The GC was run in the split mode (split ratio 16.0) with a flow of 1 mL/min of helium as a carrier gas. The MS conditions were set to have the ion source temperature at 280 °C and the interface temperature at 320 °C. The scan interval was 0.3 s, scan speed was 2000 and the mass range was 50–600 *m/z*. The collected mass spectra were matched with the NIST spectral database. Quantification of DBT and 2–HBP was performed with standard curves for both compounds prepared using authentic standards.

### 2.6 Biodesulfurization of diesel

To monitor growth of the MG1 consortium on diesel, it was grown in Erlenmeyer flasks (in triplicates) containing 90 mL of CDM with glucose (10 mM) as a carbon source and hydrodesulfurized diesel (10 %) as a sole sulfur source. All cultures were incubated at 30 °C in an orbital shaker at 180 rpm until the cultures reached the stationary phase. Samples were retrieved at intervals from all cultures for measuring the biomass (g dcw/L). To analyse the utilization of diesel hydrocarbon components, two identical sets of the MG1 cultures in CDM with glucose and diesel were prepared in triplicates. One set was immediately analyzed at day zero (beginning of the experiment) while the other set was incubated for 7 days and was analyzed at the end of the incubation period. For comparison, precultures and test cultures for the IGTS8 reference strain were prepared and processed in the same way. Uninoculated cultures were included as sterility and abiotic loss controls. All cultures were centrifuged (14,000 rpm, 15 min, 4 °C) and the diesel layer was carefully removed, re–centrifuged and analyzed by GC–MS as described above to check for degradation of the hydrocarbon components. To measure total sulfur, diesel aliquots were analyzed by X-Ray fluorescence using XOS-Sindie-7039 G3 sulfur analyzer. Cell–free culture supernatants (25 mL) were retrieved and extracted once using equal volume of GC–grade ethyl acetate. The organic phase was evaporated in a rotary evaporator, and the residue was re–dissolved in 2 mL of ethyl acetate to be analyzed by GC–MS.

## 3 Results

### 3.1 Biodesulfurization substrate spectrum of the MG1 consortium

**Fig. 1:**
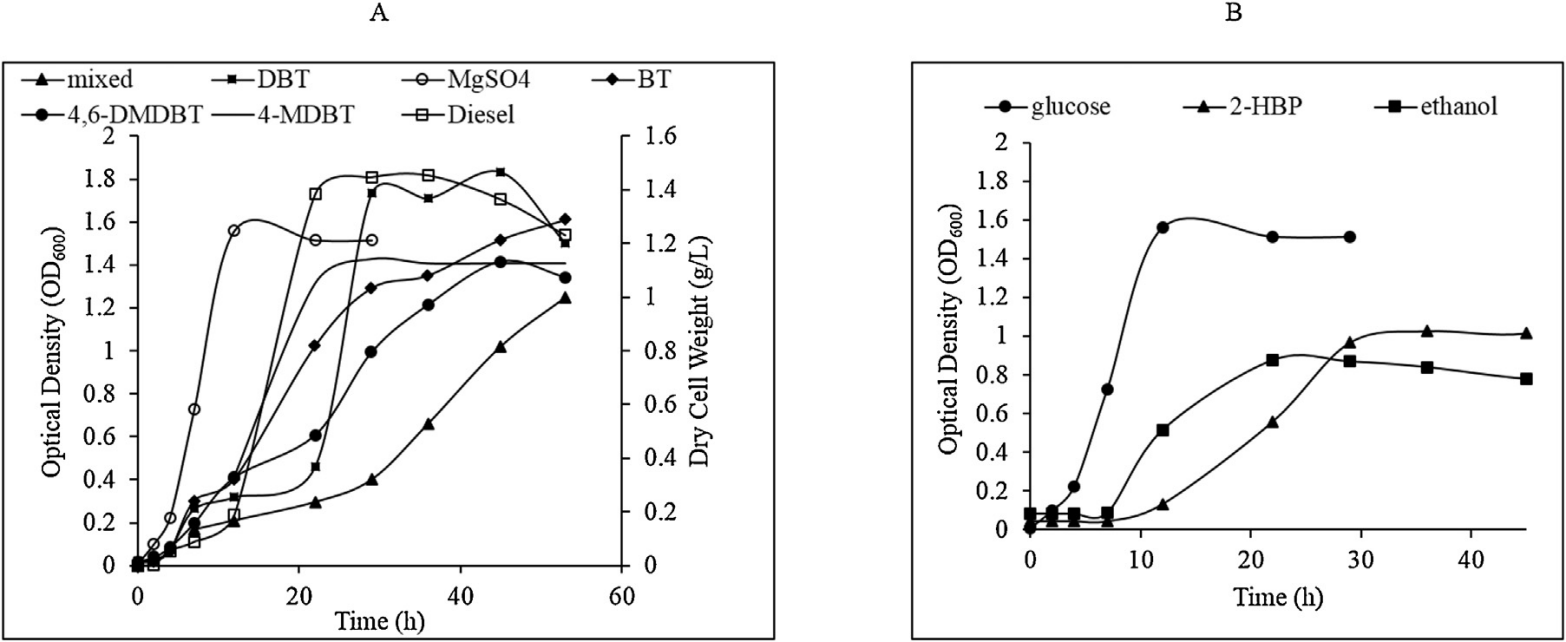
Growth profiles of the MG1 consortium in CDM with different sulfur (A) and carbon (B) sources. Growth in the diesel culture was measured as biomass dry weight.

The growth profile of the MG1 consortium varied according to the provided sulfur source (Fig. 1A, Table 2). DBS and DBT–sulfone each as a sulfur source supported only residual growth (biomass yield: 0.009 ± 0.001 – 0.01 ± 0.001 g dcw/L). MG1 did not grow on DBT, BT, 4–MDBT or 4, 6–DMDBT when provided as the carbon and sulfur source, while it grew on DBT and other thiophenic organosulfur substrates as the sole sulfur source when glucose was provided as a carbon source. The maximum specific growth rate on organosulfur substrates was attained in the DBT culture (0.078 ± .014 h^–1^), whereas the lowest was on 4, 6–DMDBT (0.053 ± 0.004 h^–1^). Among all MG1 cultures, the lowest specific growth rate (0.025 h^–1^) was observed in the culture grown on glucose as a carbon source and a mixture of the thiophenic organosulfur compounds as sulfur sources (Table 2). Comparing the growth of the MG1 consortium on organosulfur compounds with that on MgSO_4_, it was obvious that growth on MgSO_4_ was preferable (Table 2). The MG1 consortium also grew in CDM with 2-HBP or ethanol as a carbon source in the presence of MgSO_4_ as a sulfur source (Fig. 1B).

**Table 2:** Growth kinetics parameters for the MG1 cultures.

Growth substrates	Specific growth rate (*μ)* (h^–1^)	Generation time (h)	Biomass yield (g dcw/L) after 80 h
Glucose + DBT	0.078 ± .014	12.8 ± 0.29	0.65 ± 0.03
Glucose + 4–MDBT	0.055 ± 0.001	18.2 ± 0.06	0.63 ± 0.002
Glucose + 4,6–DMDBT	0.053 ± 0.004	18.9 ± 0.28	0.28 ± 0.02
Glucose + BT	0.063 ± 0.006	15.9 ± 0.25	0.30 ± 0.01
Glucose + mixed substrates (DBT + 4–MDBT + 4,6–DMDBT + BT)	0.025 ± 0.001	40 ± 0.35	0.43 ± 0.04
Glucose + 10 % Diesel	0.065 ± 0.011	15.4 ± 0.45	0.38 ± 0.02
Glucose + MgSO_4_	0.113 ± 0.001	8.8 ± 0.05	0.94 ± 0.002
Glucose + MgSO_4_ + DBT	0.133 ± 0.002	7.5 ± 0.01	1.3 ± 0.002
Ethanol + MgSO_4_	0.030 ± 0.002	33 ± 0.08	0.22 ± 0.001
2–HBP + MgSO_4_	0.068 ± 0.002	14.7 ± 0.07	± 0.002

HPLC analysis revealed that DBT was consumed and transformed to a product that co–eluted with the authentic 2–HBP when the MG1 consortium grew on DBT as a sulfur source and glucose as a carbon source (Supplementary Fig. S3). On the contrary, DBT was not consumed and 2–HBP was not produced when MG1 was grown in CDM containing a mixture of DBT and MgSO_4_ as sulfur sources and glucose as a carbon source. HPLC analysis also revealed a temporal decrease in each of 4–MDBT and 4, 6–DMDBT and the formation of polar products (Supplementary Fig. S3). DBT–sulfone and DBS were not consumed, as no decrease in their concentration over time was observed (data not shown). Interestingly, the MG1 consortium utilized 2–HBP, which is a toxic product of the 4S biodesulfurization pathway, as a sole carbon source in the presence of MgSO_4_ as a sole sulfur source and transformed 2-HBP to more polar products (Supplementary Fig. S3).

### 3.2 Composition and population dynamics of the MG1 consortium

**Fig. 2:**
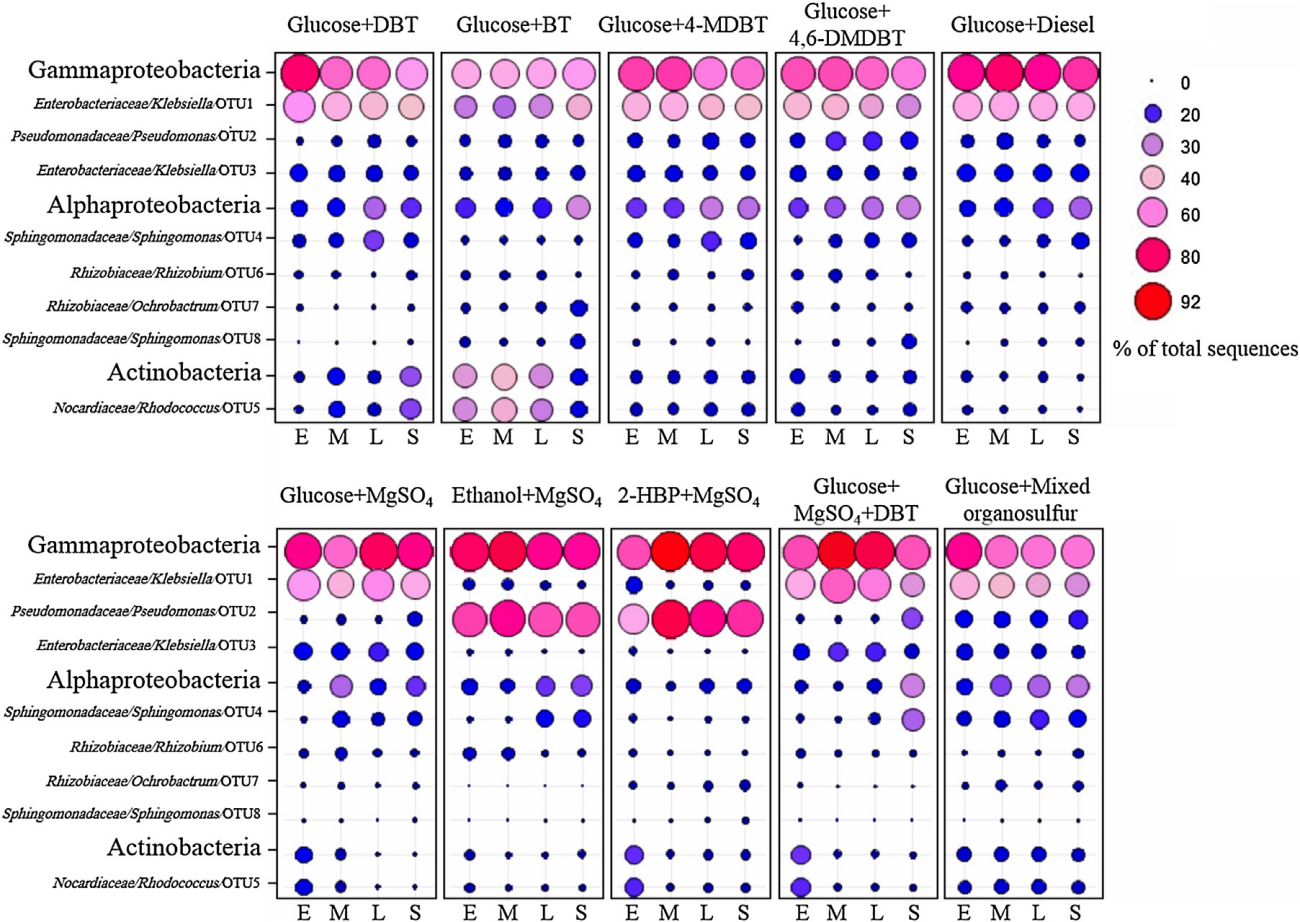
Temporal changes of major members of the MG1 consortium at different growth phases (E: early-, M: mid-, L: late-log and S: stationary phase) when grown on different carbon and sulfur sources. Note that the shown 8 OTUs made up > 92 % of the total sequences in all cultures.

**Fig. 3:**
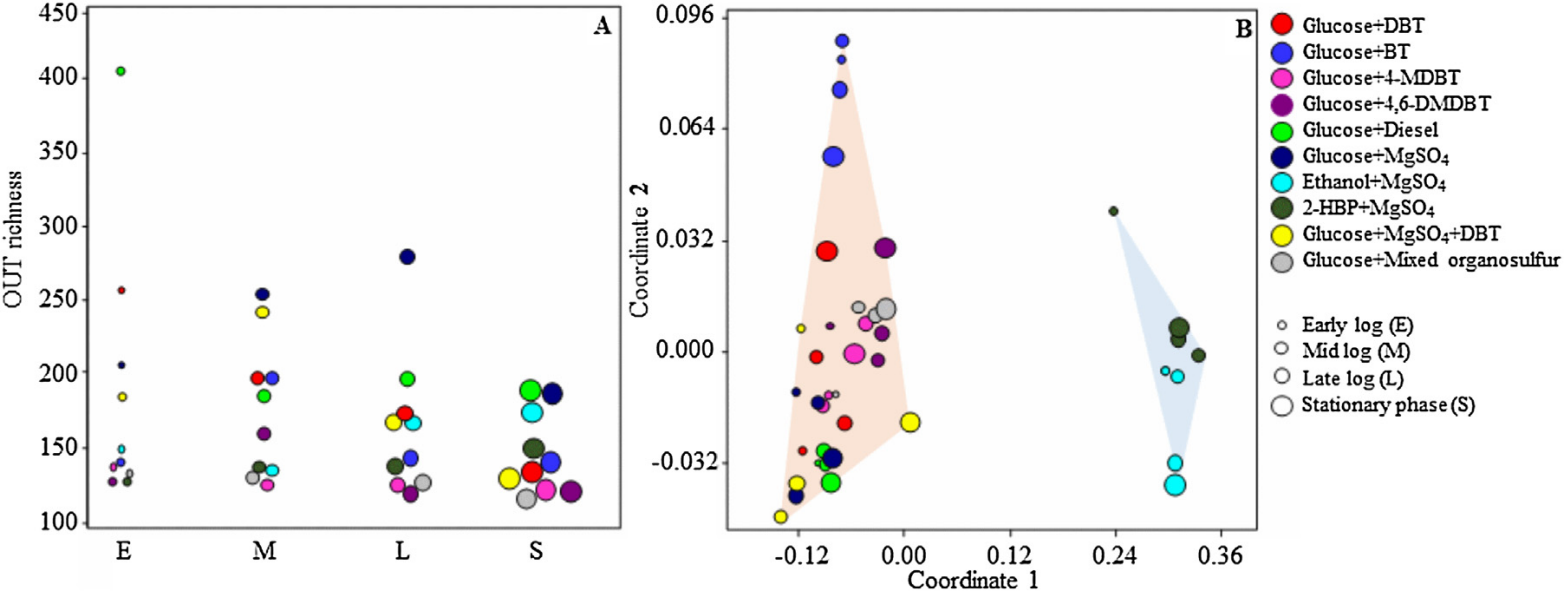
A) Estimates of OTU richness within bacterial sequences obtained from the different MG1 cultures at the different growth phases (E: early-, M: mid-, L: late-log and S: stationary phase) when grown on different carbon and sulfur sources. B) NMDS ordination plot of bacterial communities of the different MG1 cultures. Note that the bacterial communities of the cultures form two distinct clusters based on the used carbon source.

Illumina–MiSeq amplicon sequencing revealed the bacterial structure of the MG1 consortium and shifts in its composition at the different stages of growth on various sulfur and carbon sources (Table 1). The number of OTUs (operational taxonomic units), calculated using subsets with the same number of sequences for all samples, did not exceed 403 OTUs per sample, although the number of obtained sequences was between 128,156 and 418,871 reads per sample (Supplementary Tables S2 and S3). Rarefaction curves showed that all samples reached a maximum yield of OTUs (Supplementary Fig. S4). The OTUs richness varied between the different cultures and at the different growth phases (Fig. 2). At the early-log phase, the highest OTU richness was observed in the MG1 consortium growing on glucose with either diesel (400 OTUs), DBT (255 OTUs), MgSO_4_ (205 OTUs) or a mixture of MgSO_4_ and DBT (182 OTUs). At the mid-log phase, the glucose + MgSO_4_ cultures with and without DBT had the highest OTUs number of 254 and 242, respectively, followed by the MG1 cultures on glucose with DBT, BT or diesel. The MG1 cultures on glucose and either MgSO_4_ or diesel still maintained the highest number of OTUs at the late-log and stationary phases. The OTUs richness did not vary much (121–155 OTUs) at the different growth phases of the MG1 consortium grown on glucose + 4-MDBT, 2-HBP + MgSO_4,_ and glucose + mixed organosulfur compounds. When variations in bacterial community composition of the different MG1 cultures were visualized in a two-dimensional space using multivariate analyses of OTUs (Fig. 3), the bacterial communities were clearly segregated in two separate clusters based on the used carbon source (ANOSIM *R* = 0.66, *P* = 0.0001). Cultures with MgSO_4_ as the sulfur source and either ethanol or 2-HBP as a carbon source clustered together and their structure was clearly different from those containing glucose as the carbon source, regardless of the used sulfur source. Within the glucose cultures’ cluster, those with BT as a sulfur source were most distant from the rest.

Alphaproteobacteria, Gammaproteobacteria and Actinobacteria constituted more than 96 % of the total number of sequences in all cultures of the MG1 consortium. Sequences belonging to Gammaproteobacteria made up between 46 % and 92 % of the total number of sequences with the lowest relative abundance in the glucose + BT cultures. Most gammaproteobacterial sequences were distributed between the two genera *Klebsiella* and *Pseudomonas*. *Klebsiella* exhibited higher relative abundance than *Pseudomonas* in all cultures with glucose; however, when either ethanol or 2-HBP was used as a carbon source, *Pseudomonas* uniquely dominated the MG1 consortium (50–85 % of total sequences). Alphaproteobacteria made up 5 %–32 % of the total number of sequences in all cultures of the MG1 consortium, but with relatively higher proportions during the late-log and stationary growth phases compared with the early- and mid-log phases. The majority of alphaproteobacterial sequences were affiliated to the genera *Sphingomonas*, *Rhizobium* and *Ochrobactrum*. The distribution of these genera varied among the different cultures and at the different growth phases (Fig. 2). Most of the actinobacterial sequences belonged to the genus *Rhodococcus*, which exhibited a sequence proportion of as low as 1% and as high as 36 % of the total number of sequences, with higher proportions during the early-log phases of growth. The *Rhodococcus* relative abundance decreased with time in most cultures, except in the glucose + DBT cultures where it increased to reach 25 % of total sequences during the stationary phase. The proportions of Actinobacteria in glucose cultures with either 4-MDBT, 4, 6-DMDBT or mixed organosulfur substrates did not change much at the different growth phases. The highest occurrence of Actinobacteria was detected in the glucose + BT cultures, reaching 36 % of all sequences during the mid-log phase but then decreased to 15 % at the stationary phase.

### 3.3 The biodesulfurization pathway adopted by the MG1 consortium

**Fig. 4:**
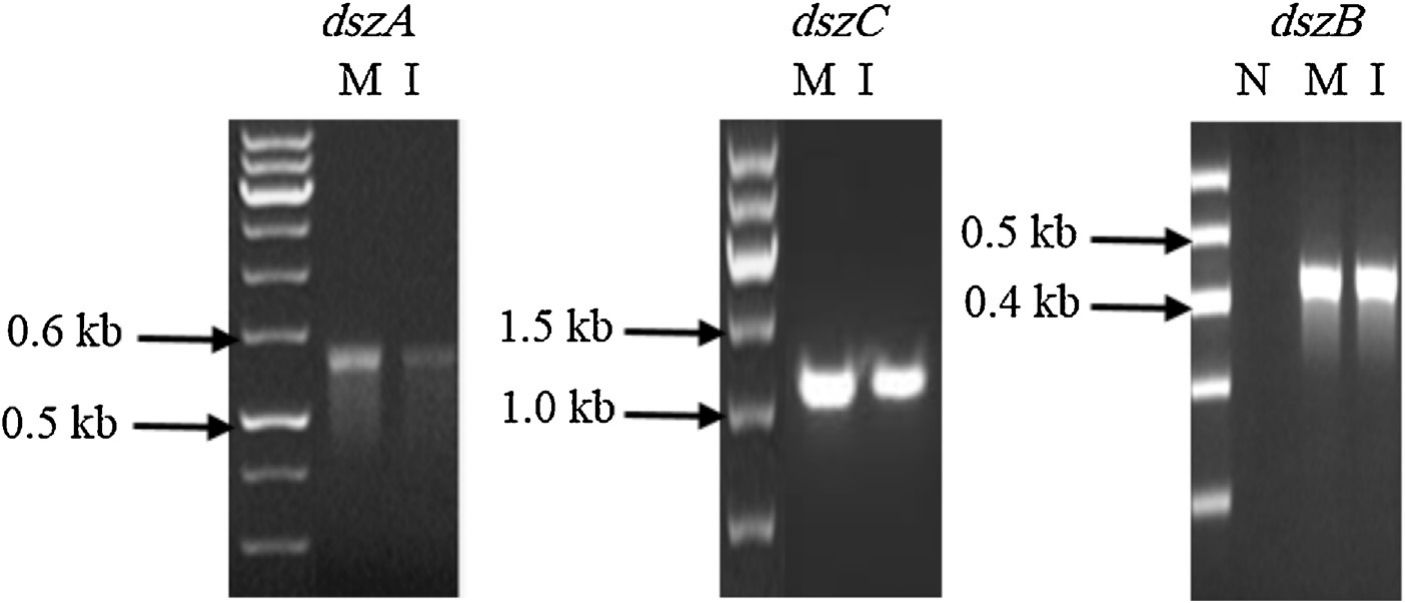
Agarose gel images showing the amplification of fragments of the 4S biodesulfurization pathway genes using total genomic DNA isolated from cultures of the MG1 consortium grown on DBT as a sole sulfur source and glucose as a carbon source. N: negative control, M: MG1, I: IGTS8.

**Fig. 5:**
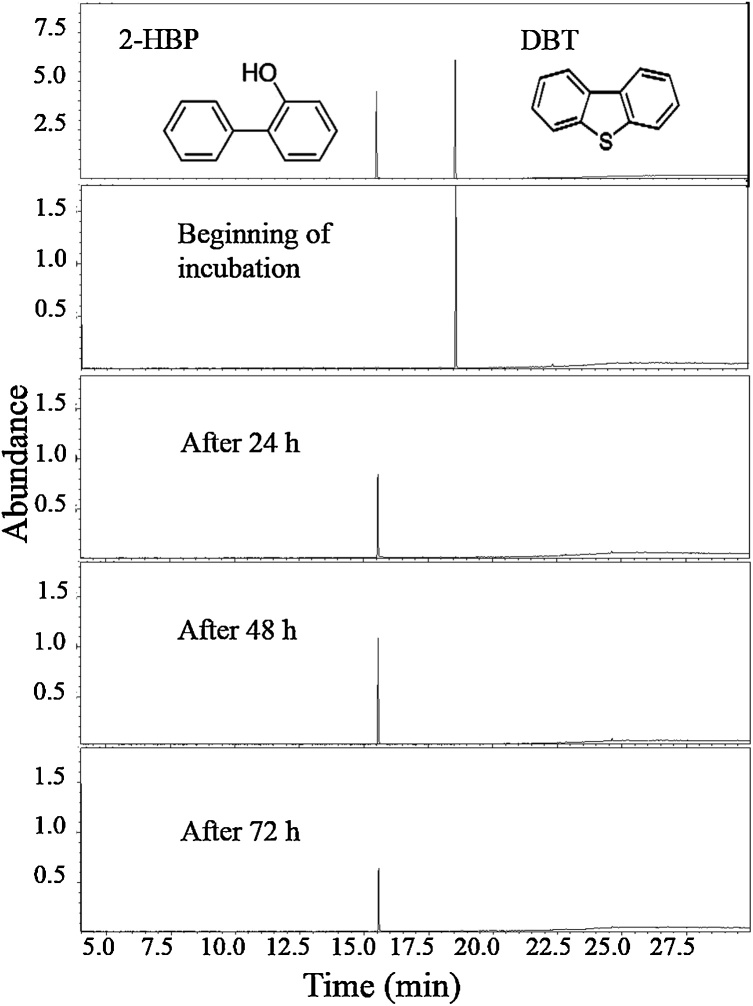
Total ion chromatograms of ethylacetate extract from MG1cultures grown in CDM containing glucose as a carbon source and DBT as a sole sulfur source. Upper panels show chromatograms of authentic samples.

**Fig. 6:**
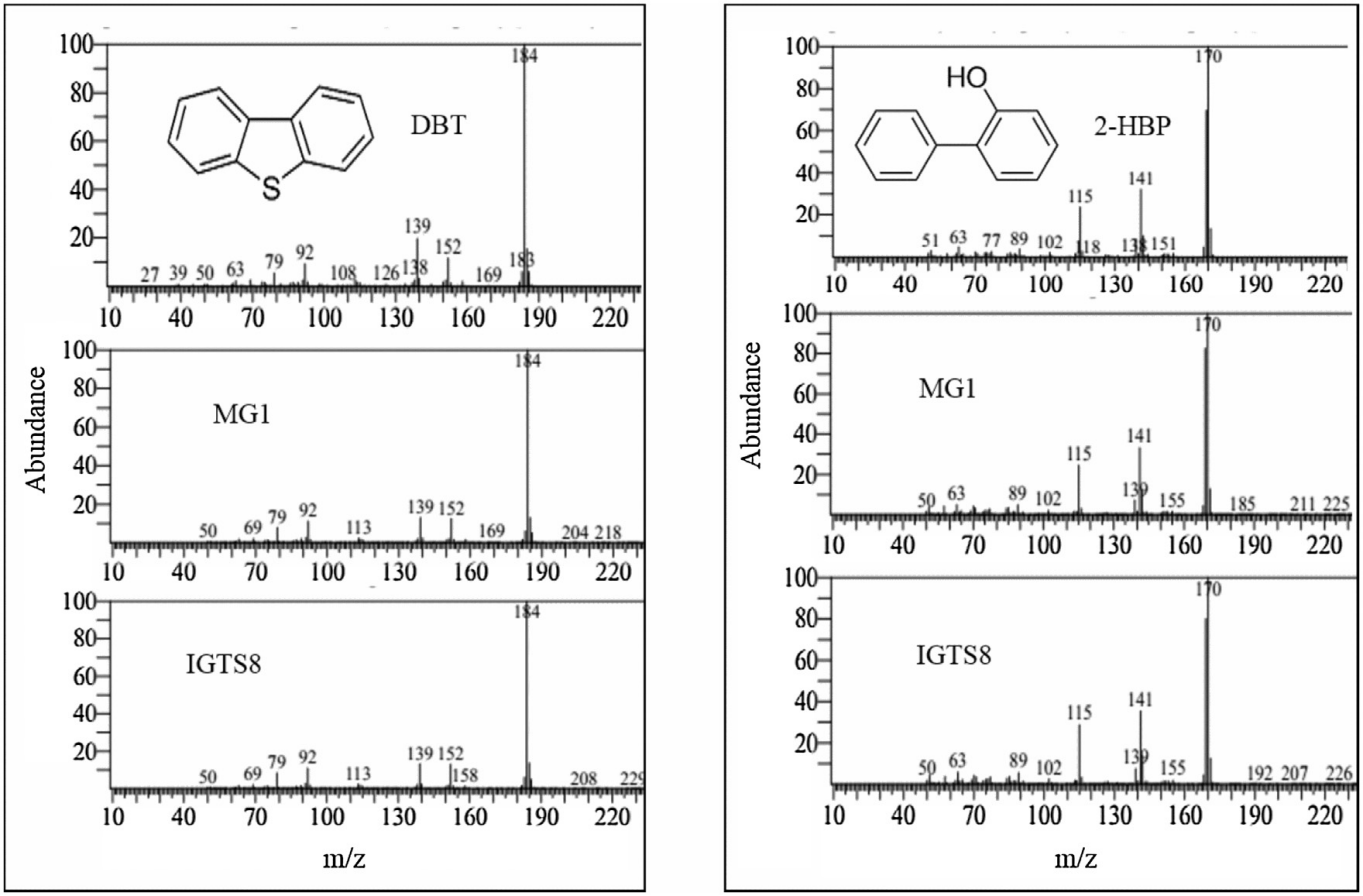
Mass spectra of 2–HBP and DBT detected in the IGTS8 and MG1 cultures grown in CDM containing glucose as a carbon source and DBT as a sole sulfur source. Upper panels show mass spectra of authentic samples.

Fragments of *dszB* (0.42 kb)*, dszC* (1.25 kb) and *dszA* (0.547 kb) genes of the 4S pathway could be PCR–amplified using DNA isolated from a DBT-grown culture of MG1 (Fig. 4). In addition, GC–MS analysis of DBT–grown MG1 cultures revealed two main peaks at 18.5 min and 15.5 min, which were identical to the retention time of authentic DBT and 2-HBP, respectively (Fig. 5). The mass spectra of compounds under the 18.5 min and the 15.5 min peaks were identical to those of authentic DBT and 2–HBP with the characteristic molecular ion at 184 *m/z* and 170 *m/z*, respectively (Fig. 6). They were also identical to mass spectra of compounds detected under the corresponding peaks in total ion chromatograms (not shown) from DBT cultures of the model biodesulfurizing strain IGTS8 (Fig. 6). The concentration of DBT reached 0.0001 mM in the MG1 cultures and 0.0002 mM in the IGTS8 cultures after 24 h of incubation and in both cultures DBT was consumed within 72 h of incubation. The concentration of 2–HBP in the MG1 culture was 0.041 mM after 24 h and increased to 0.056 mM after 72 h of incubation. In the IGTS8 cultures, a similar pattern was observed where the 2–HBP concentration was 0.03 mM after 24 h and increased to 0.055 mM after 72 h of incubation.

### 3.4 Biodesulfurization of hydrodesulfurized diesel

**Fig. 7:**
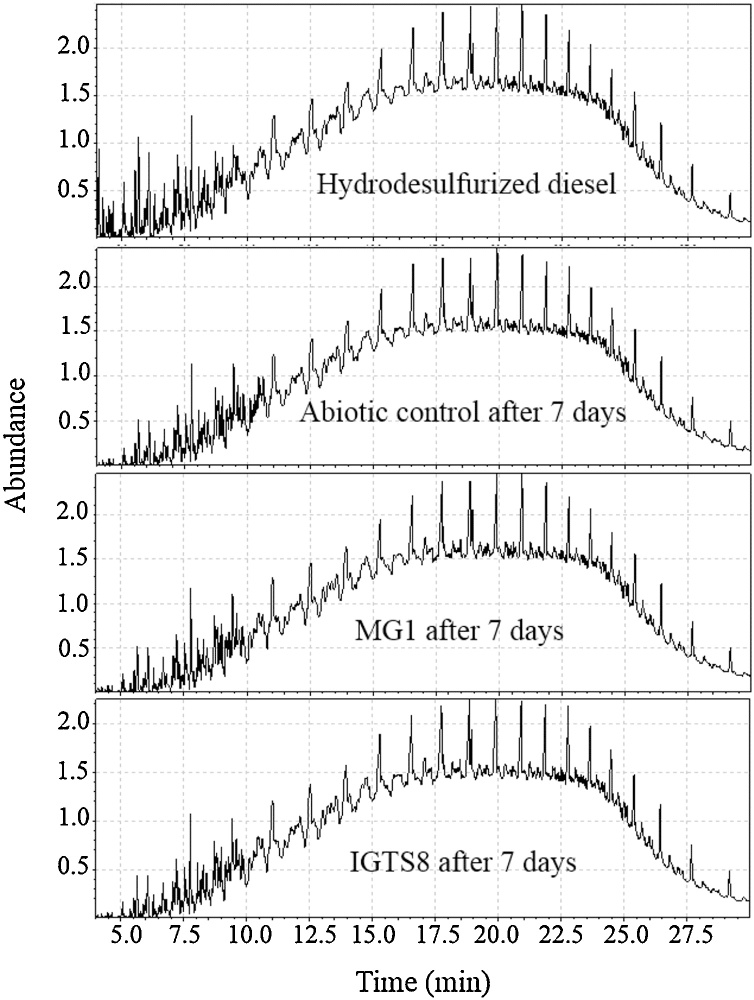
Total ion chromatograms of hydrodesulfurized diesel before and after biological treatment with MG1 and IGTS8 cultures in CDM containing glucose as a carbon source and hydrodesulfurized diesel as a sole sulfur source.

X-Ray fluorescence analysis revealed that the MG1 consortium and the IGTS8 strain reduced the sulfur content of diesel by 25 % within 7 days of incubation in biphasic batch cultures as compared to diesel samples analyzed at the beginning of the experiment. Furthermore, GC–MS analysis revealed no remarkable differences between the as–received (original), abiotically treated, and the biologically treated diesel, considering the resolvable peaks (Fig. 7). Ethyl acetate extracts of cell–free culture supernatants contained several hydrocarbon peaks, which apparently originated from the diesel phase (data not shown). The biodesulfurization product 2-HBP could not be detected either in biotreated diesel or in the ethyl acetate extracts of the culture aqueous phase. Similar results were obtained for cultures of the IGTS8 strain grown under the same conditions as the MG1 consortium (not shown).

## 4 Discussion

### 4.1 Biodesulfurization phenotype of the MG1 consortium

Growth on and utilization of hydrodesulfurized diesel and several diesel-born thiophenic compounds as sole sulfur sources confirmed the biodesulfurization phenotype of the MG1 consortium. These results show the broad substrate spectrum of MG1, which is a desirable trait in view of industrial application [16,19]. This is particularly important for DBT, BT and their alkylated derivatives, which constitute the major fraction of sulfur in crude oil and diesel [1,30] and, therefore, are the target for deep desulfurization since they resist the conventional hydrodesulfurization [31]. In contrast, the majority of known biodesulfurizing microbes have a narrow substrate spectrum where they can utilize either DBT or BT as a sulfur source [1,19,32]. It is well documented that easily bioavailable sulfur sources, such as inorganic sulfate, strongly inhibit biodesulfurization via the 4S pathway [1,8], which explains why the MG1 consortium in cultures containing a mixture of DBT and MgSO_4_ did not consume DBT.

The growth profile (specific growth rates, generation times, and biomass yields) variations between the MG1 cultures grown on different sulfur sources could be explained by differences in the biodesulfurization susceptibility of the tested substrates [16,33]. It is known that 4, 6–DMDBT is more recalcitrant than DBT toward biodesulfurization via the 4S pathway [33]. It can be also proposed that the different sulfur substrates were utilized by different populations from the same consortium (see later, data from this study). A mixed culture consisting of the genera *Sphingobacterium, Klebsiella, Pseudomonas, Stenotrophomonas, Arthrobacter*, *Mycobacterium* and *Rhodococcus* utilized DBT, BT, 4–MDBT and 4, 6–DMDBT with different growth patterns in terms of specific growth rate, generation time, and biomass yield [17]. Also, Garrido-Sanz et al. [34] reported variations in growth patterns and yields for a diesel-degrading bacterial consortium grown on different hydrocarbons. The lowest specific growth rate observed for the MG1 consortium when grown on a mixture of organosulfur substrates may be due to competitive inhibition by the substrates, which reduces the biodesulfurization rate and leads, consequently, to slower growth. In a study on *Mycobacterium* sp. ZD–19, Zhang et al. [35] noted that biodesulfurization decreased in a culture containing a mixture of DBT, 4–MDBT, and 4, 6–DMDBT as compared to cultures containing any of these sulfur sources individually. Similarly, Kobayashi et al. [36] reported that the biodesulfurization activity of *R. erythropolis* KA2-5–1 with alkyl DBT substrates was reduced when the different compounds were mixed, as compared to systems having them separately. In both studies, the apparent competitive inhibition by the substrates was predictable by a Michaelis–Menten competitive inhibition model.

The observed decrease in the total sulfur content of MG1-treated diesel highlights the capabilities of the MG1 consortium as a fuel–biodesulfurizing culture. The result is in line with the presence of potential biodesulfurizers among the MG1 community, in particular *Rhodococcus*, *Klebsiella* and *Sphingomonas* spp. [16,37]. It was also interesting to see that the diesel matrix was not significantly altered by extensive biodegradation of the hydrocarbon components by MG1. It appears that the MG1 consortium can perform a selective attack on the thiophenic compounds of diesel without consuming the hydrocarbon components. It is, however, important to consider that glucose was provided in the diesel cultures as a carbon and energy source, which probably prevented (by catabolite repression) the MG1 members from attacking the diesel hydrocarbons. In the same context, it is worth noting that the incubation period was only 7 days, which was probably too short for the MG1 consortium to start consuming the diesel hydrocarbons, particularly in the presence of a preferred carbon source like glucose. Biological treatment of diesel with microbial consortia was extensively studied in the context of biodegradation and bioremediation [34,[38], [39], [40]]. To the contrary, biological treatment of diesel or diesel-born organosulfur compounds with microbial consortia for biorefining processes such as biodesulfurization has been very rarely reported. Previously, a consortium of *R. erythropolis* DS–3 and *Gordonia* sp. C–6 utilized hydrodesulfurized diesel as a sulfur source (86 % reduction in sulfur content) by attacking many of the diesel-born thiophenic compounds [41].

Many studies with axenic cultures reported biodesulfurization activity on diesel that is similar to, lower or higher than that achieved by the MG1 consortium. For instance, while the MG1 consortium reduced the sulfur content of hydrodesulfurized diesel by 25 % within 7 days, *Sphingomonas subarctica* T7b reduced it by 59 % after 36 h [42]. A growing culture of *Rhodococcus* sp. ECRD-1 reduced the sulfur content of a middle distillate fraction by 8.1 % after 168 h [43]. It is, however, important to pay attention when comparing biodesulfurization rates or activities of different microbes reported in different studies, mainly because of the differences in the applied biodesulfurization conditions (oil/water ratio, incubation time, temperature, biomass load, agitation speed) [19]. In the current study, since the same conditions were maintained in the diesel biodesulfurization experiments for both the MG1 consortium and the reference strain IGTS8, we could reliably conclude that the biodesulfurization activity of the MG1 consortium was similar to that of the model IGTS8 strain.

### 4.2 Evidence for the 4S pathway

Several lines of evidence confirmed DBT biodesulfurization by the MG1 consortium via the 4S pathway. GC–MS analysis gave a direct evidence for the conversion of DBT to 2–HBP, which is a key product of the 4S biodesulfurization pathway [4,8,16]. In addition, results of GC–MS analysis of the MG1 cultures were identical to those of the reference strain IGTS8, which is known to use the 4S pathway [1,44]. The detection of the 4S pathway genes in the genomic DNA extracted from the DBT–grown MG1 cultures together with the presence of *Rhodococcus* spp., the most common biodesulfurizers via the 4S pathway [1,8,16], in the MG1 consortium further corroborate the functioning of the 4S pathway. These evidences are further backed by lack of growth on DBT, and other tested thiophenic compounds, as a carbon and energy source, which excludes the presence of a destructive or mineralization pathway amongst the MG1 community members [8,16]. In fact, we found a few studies on biodesulfurization by mixed cultures in the literature and the 4S pathway was reported only in some [17,45].

It is well known that 2-HBP, the final product of the 4S biodesulfurization pathway in the majority of biodesulfurizing bacteria, exhibits inhibitory effects on growth and biodesulfurization enzymes [1,46]. Thus, it is beneficial for biodesulfurizing cultures to eliminate this toxic compound via biotransformation to other nontoxic products [12,13]. Accordingly, the ability of the MG1 consortium to grow on 2-HBP indicates its potential to detoxify this compound, which is another desirable trait in view of commercialization.

### 4.3 Population dynamics in the MG1 consortium

Illumina–MiSeq sequencing provided information on the composition of the MG1 consortium and shifts accompanying growth on different carbon and sulfur sources. However, this technique does not necessarily unravel the role of each member of the consortium in the biodesulfurization process, unless each member of the consortium is isolated and studied in pure cultures. Even this does not guarantee revealing the exact role since bacteria might behave differently in consortia than when grown individually [47,48]. Nonetheless, potential roles could be proposed based on changes in the relative abundance of members within the consortium since the increase in abundance of a certain community member suggests a potential role in the underlying process [34,[49], [50], [51]]. According to Sydow et al. [40], the following two determinants may dictate the changes in the relative abundance of a member in a microbial consortium when challenged with different growth substrates; (i) the ability of that member to grow on and utilize the provided substrate, (ii) the growth kinetics on that substrate. Although Sydow et al. [40] were addressing hydrocarbon degradation in their study, we argue that their assumptions can be extrapolated to explain sulfur source-driven community dynamics.

Indeed, previous research has shown that biodesulfurizing consortia may comprise both biodesulfurizing and non-biodesulfurizing members [17,52], which could also be the case in the MG1 consortium given the high number of detected OTUs. The non-biodesulfurizing community members might contribute by providing essential nutrients, vitamins, biosurfactants [20,21] or removing toxic intermediates [53]. They can also flourish by feeding on biodesulfurization intermediates or debris of dead cells [54]. The non–desulfurizing members definitely need sulfur for growth, which can be accessed from sulfate released from the organosulfur source by the actual biodesulfurizers [22].

Analysis of the MG1 community structure revealed both temporal and substrate-driven compositional shifts. Based on the snapshots taken for the MG1 community at different growth phases, it appears that succession of populations occurred, which can be reconciled particularly for cultures containing diesel and the mixed organosulfur substrates as these multi-component substrates might need successive communities at different growth phases. Temporal shifts in the structure of hydrocarbon-degrading microbial communities were reported [34,55,56]. For the sake of comparison, the composition of the MG1 consortium in the glucose+MgSO_4_ culture, which was dominated by *Klebsiella* and *Sphingomonas* spp., was considered as the base line for defining compositional shifts in all other MG1 cultures. It is worth noting that among the MG1 community members only *Rhodococcus* and *Klebsiella* spp. were reported to utilize DBT via the 4S biodesulfurization pathway [8,16]. In the DBT cultures, persistence of *Klebsiella* spp. and the increase in the relative abundance of *Rhodococcus* spp., compared to the MgSO_4_ culture, highlight the response of the MG1 community to the stress of sulfur limitation imposed by DBT and suggest that *Rhodococcus* spp. in this culture play a key role in DBT biodesulfurization. This is in accordance with the fact that many DBT biodesulfurizers are *Rhodococcus* spp. [1,7,8,16]. It can be inferred that *Rhodococcus* spp. are also the key biodesulfurizers in the BT-shaped MG1 consortium based on their higher relative abundance even when compared to the DBT cultures, which is the reason for the unique clustering of the BT–grown community as revealed by the NMDS analysis. In addition, the increased relative abundance of *Sphingomonas* spp. in both DBT and BT cultures as compared to the MgSO_4_ cultures indicates a potential role of these bacteria in the utilization of these substrates [42,57]. Although *Rhodococcus* spp. became less dominant in the 4–MDBT and 4, 6–DMDBT cultures, as compared with the cultures on DBT and BT, we still believe that they are involved in biodesulfurization of the alkylated DBT derivatives but were growing slower due to the methyl groups [58,59]. In the culture containing a mixture of thiophenic compounds, *Klebsiella, Pseudomonas* and *Rhodococcus* spp. appear to be the key players since they dominated the MG1 community through all growth phases.

Since DBT was not consumed in the DBT + MgSO_4_ culture, it can be concluded that the abundant members of MG1 utilized the inorganic sulfate as a sulfur source, which is in line with the fact that the expression of the 4S biodesulfurization enzymes is strongly repressed in the presence of easily bioavailable sulfur sources, such as inorganic sulfate [1,16].

To the best of our knowledge, there is no published data on the community structure dynamics of consortia during diesel biodesulfurization. Based on the relative abundance of the MG1 consortium members in the diesel cultures, it can be proposed that the most abundant members of the diesel–grown MG1 community, namely, *Klebsiella, Pseudomonas* and *Sphingomonas* are the actual biodesulfurizers. Different members of the diesel–grown MG1 community can be jointly involved in the biodesulfurization process to enable the community to thrive by utilizing the different organosulfur compounds present in diesel [41]. A direct role of *Pseudomonas* spp. in biodesulfurization of the diesel-born thiophenic compounds may be debated based on the fact that, to date, no native *Pseudomonas* strains have been reported to harbour the 4S pathway, and the known biodesulfurizing *Pseudomonas* spp. that could utilize the 4S pathway are laboratory-designed recombinant strains [8,60]. Accordingly, we argue that *Pseudomonas* spp. in the MG1 consortium were thriving by utilization of biodesulfurization products such as 2-HBP [61,62].

The remarkable compositional shifts in the MG1 consortium when provided with different carbon sources show the key role of *Pseudomonas* spp. in the utilization of ethanol and 2–HBP [61,62]. The NMDS analysis confirmed the uniqueness of the ethanol– and 2–HBP– shaped MG1 consortia, which is obviously due to the unparalleled dominance of *Pseudomonas* spp. as compared to all glucose–containing cultures. Since *Pseudomonas* spp. in the DBT–shaped MG1 community constituted an important fraction (9% on average, reached 14 % at the mid–log phase), they could be involved in the degradation of 2–HBP resulting from biodesulfurization of DBT via the 4S pathway. In support of this hypothesis, HPLC analysis confirmed 2–HBP consumption by MG1. The ability of MG1 to degrade/transform 2–HBP to none or less toxic products is a desirable trait that could relieve the biodesulfurizing community from the negative impact of 2–HBP on growth and biodesulfurization activity [13,46]. It appears that the contribution of ethanol to the structure of the MG1 community was masked by that of other major nutrients (carbon and sulfur source). Ethanol does not seem to have played a decisive role in shaping the MG1 community in the different cultures because cultures with glucose and different organosulfur substrates had different relative abundances of the main genera like *Klebsiella, Pseudomonas* and *Rhodococcus*, although these cultures had the same concentration of ethanol.

The results reported in this study provide a ground for future studies on biodesulfurizing mixed cultures which can implement an integrated approach combining metaproteomics and metagenomics to enable better understanding of the structure–function dynamics of the biodesulfurizing consortia. In particular, structural and functional resistance and resilience of the biodesulfurizing mixed cultures need in–depth investigations. Moreover, synthetic biology and metabolic engineering studies should be engaged to tailor–design or develop compositionally optimized microbial consortia for a commercially viable fuel desulfurization bioprocess. Notwithstanding the relatively low sulfur removal rate, we believe that microbial consortia deserve further optimization and bioengineering efforts.

## 5 Conclusions

The main conclusion drawn from our findings is that the type of sulfur and carbon source can affect the structure of a diesel-biodesulfurizing consortium. The observed temporal compositional shifts were manifested as variations in the relative abundance of key members of the microbial consortium, which reflected the presence of both biodesulfurizing and potentially non-biodesulfurizing strains. As compared to axenic cultures, biodesulfurizing consortia may have the advantage of better tolerance to the toxic biodesulfurization products such as 2–HBP, in addition to the broader substrate spectrum.

## Declaration of Competing Interest

The authors have no competing interests.

## Funding

The authors acknowledge the financial support provided by 10.13039/100009606Arabian Gulf University (Grant no. LS_WE_2017).

## CRediT authorship contribution statement

**Maysoon Awadh:** Investigation, Methodology, Writing - original draft. **Huda Mahmoud:** Supervision, Investigation, Writing - original draft. **Raeid M.M. Abed:** Investigation, Data curation, Writing - review & editing. **Ashraf M. El Nayal:** Methodology. **Nasser Abotalib:** Methodology. **Wael Ismail:** Conceptualization, Investigation, Funding acquisition, Supervision, Project administration, Writing - original draft, Writing - review & editing.
