# Supplementary material for: Diesel-born organosulfur compounds stimulate community re-structuring in a diesel-biodesulfurizing consortium
Source: Biotechnol Rep (Amst). 2020 Nov 23;28:e00572. doi: 10.1016/j.btre.2020.e00572 (PMC7749429; doi:10.1016/j.btre.2020.e00572)
Supplement: Supplementary file 1 [file mmc1.docx]

**Biotechnology Reports**

**Supplementary Material**

**Diesel-Born Organosulfur Compounds Stimulate Community Re-structuring in a Diesel-Biodesulfurizing Consortium**

Maysoon Awadh^1^, Huda Mahmoud^2^, Raeid M. M. Abed^3^, Ashraf M. El Nayal^1^, Nasser Abotalib^1^, Wael Ismail^*1^

^1^Environmental Biotechnology Program, Life Sciences Department, College of Graduate Studies, Arabian Gulf University, Manama, Kingdom of Bahrain

^2^Department of Biological Sciences, Faculty of Science. Kuwait University, Kuwait

^3^Biology Department, College of Science, Sultan Qaboos University, Muscat, Oman

^*^Corresponding author: Environmental Biotechnology Program, Life Sciences Department, College of Graduate Studies, Arabian Gulf University, Bahrain, Tel: +97336146948; Fax: +97317239664, E-mail: [waelame@agu.edu.bh](mailto:waelame@agu.edu.bh)


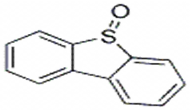

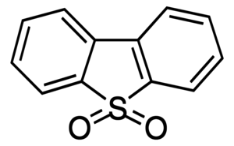

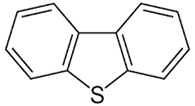


**(DszC)**

**(DszC)**

**(DszC)**

**DBT sulfoxide DBT sulfone**

**DszA**


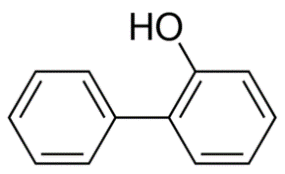

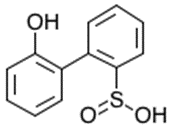


**DszB**

**SO_3_^2–^  +**

**2-HBP HPBS**

Fig. S1: The 4S biodesulfurization pathway. HPBS: hydroxyphenylbenzenesulfinate, 2-HBP: 2-hydroxybiphenyl.


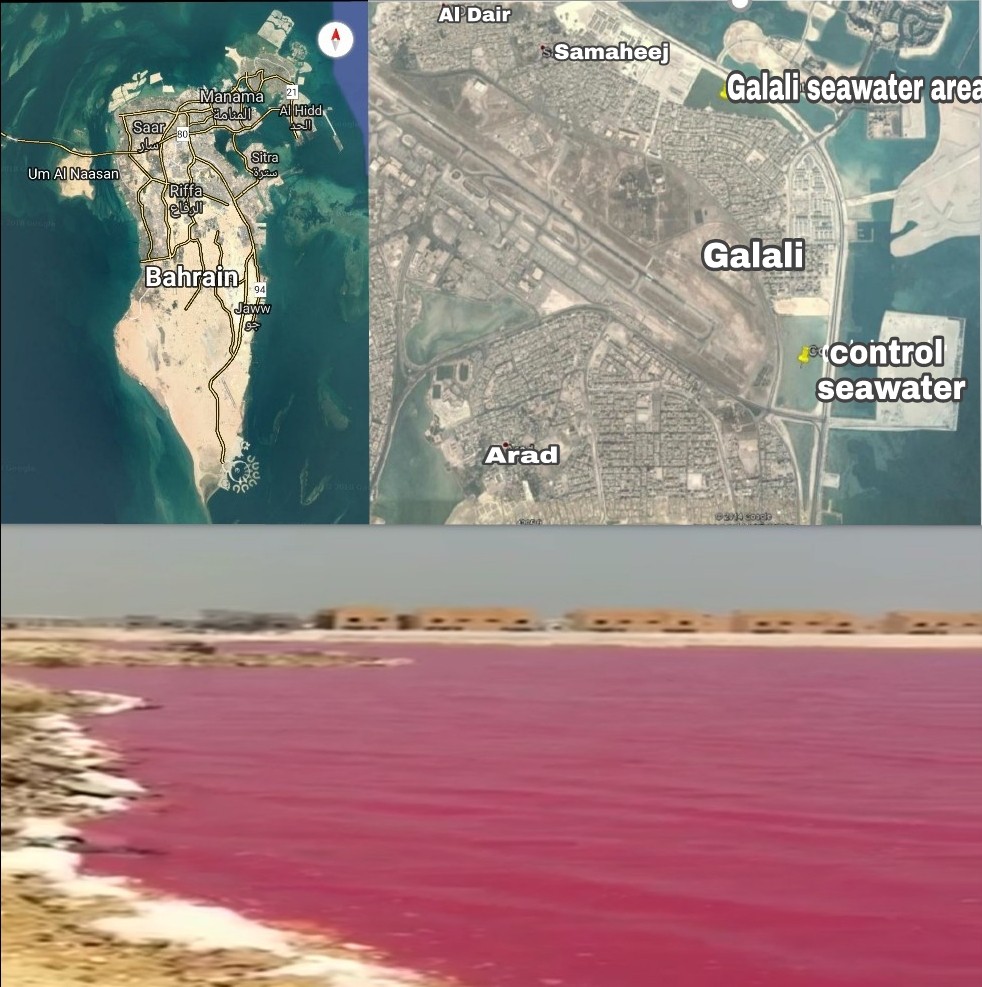


Fig. S2: Location of the rose lagoon at Galali-Muharraq Island at the north east of Bahrain. The lagoon appeared in a reclaimed seashore area from March to May 2014. The lagoon had offensive rotten egg odor.


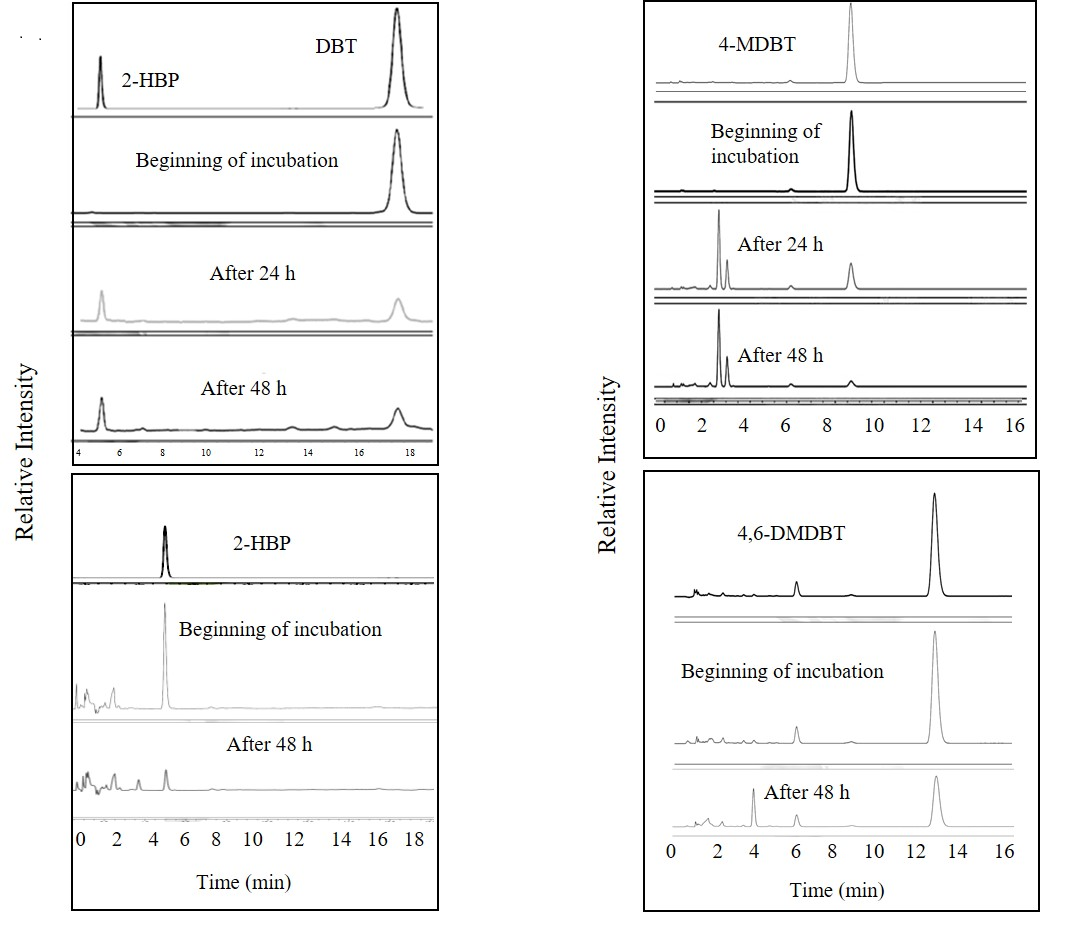


Fig. S3: HPLC analysis showing the utilization of 2-HBP and organosulfur compounds by the MG1 consortium. Upper panels show chromatograms of authentic samples.

Fig. S4: Calculated rarefaction curves of observed OTUs (sequences that have 97% similarity are defined as one OTU) richness in the different cultures of MG1 consortium on different carbon and sulfur sources (see table S3 for sample identification).

Table S1: Composition of the chemically defined medium (CDM)

| Stock solution | Stock solution  concentration | Volume added (mL) per L of medium |
| --- | --- | --- |
| K–phosphate buffer  (pH 7.2)  K_2_HPO_4_  KH_2_PO_4_ | **(1 M)**  140 g/L  27 g/L | 50 mL |
| NH_4_Cl | 1 M (54 g/L) | 10 mL |
| Sulfur free–deionized water |  | 929 mL |
|  | | |
| CaCl_2_.2H_2_O | 0.3 M | 1 mL |
| MgCl_2_.6H_2_O* | 1 M | 1 mL |
| FeCl_2_.4H_2_O | 0.01M | 1 mL |
| Sulfur–free trace elements  ZnCl_2_.7H_2_O  MnCl_2_.4H_2_O  CuCl_2_  CoCl_2_.6H_2_O  Na_2_MoO_4_.2H_2_O  NiCl_2_.6H_2_O  H_3_Bo_3_ | 70 mg/L  100 mg/L  20 mg/L  200 mg/L  40 mg/L  20 mg/L  20 mg/L | 1 mL |
| Vitamins Solution  Cyanocobalamine (B12)  Pyridoxamin–HCl  Thiamin–HCl (B1)  Nictotinic acid  *p*–Aminobenzoic acid  Biotin  Ca–Pantothenate | 25mg/250 mL  75mg/250 mL  50 mg/250 mL  50 mg/250 mL  40 mg/250 mL  40 mg/250 mL  25 mg/250 mL | 1 mL |
| MgSO_4_.7H_2_O ** 1 M | | 1 mL |
| Glucose solution | 2M | 5 mL |
| * When an organosulfur compound was added as a sole sulfur source, MgCl_2_ was added instead of MgSO_4_  ** The sulfur source was either MgSO_4_ or an organosulfur compound | | |

Table S2: Illumina-MiSeq sequencing and diversity estimators of the different MG1 consortia (see Table S3)

Table S3: Description of the MG1 cultures used in MiSeq analysis

| **Sample ID** | **Substrate ***  **(growth phase)** |  | **Sample**  **ID** | **Substrate**  **(growth phase)** |
| --- | --- | --- | --- | --- |
| S1 | DBT (EL) |  | S21 | MgSO_4_ (EL) |
| S2 | DBT (ML) |  | S22 | MgSO_4_ (ML) |
| S3 | DBT (LL) |  | S23 | MgSO_4_ (LL) |
| S4 | DBT (SP) |  | S24 | MgSO_4_ (SP) |
| S5 | BT (EL) |  | S25 | Ethanol (EL) |
| S6 | BT (ML) |  | S26 | Ethanol (ML) |
| S7 | BT (LL) |  | S27 | Ethanol (LL) |
| S8 | BT (SP) |  | S28 | Ethanol (SP) |
| S9 | 4–MDBT (EL) |  | S29 | 2–HBP (EL) |
| S10 | 4–MDBT (ML) |  | S30 | 2–HBP (ML) |
| S11 | 4–MDBT (LL) |  | S31 | 2–HBP (LL) |
| S12 | 4–MDBT (SP) |  | S32 | 2–HBP (SP) |
| S13 | 4,6–DMDBT (EL) |  | S33 | MgSO_4_+DBT (EL) |
| S14 | 4,6–DMDBT(ML) |  | S34 | MgSO_4_+DBT (ML) |
| S15 | 4,6–DMDBT (LL) |  | S35 | MgSO_4_+DBT (LL) |
| S16 | 4,6–DMDBT (SP) |  | S36 | MgSO_4_+DBT (SP) |
| S17 | Diesel (EL) |  | S37 | Mixed organosulfur  (EL) |
| S18 | Diesel (ML) |  | S38 | Mixed organosulfur (ML) |
| S19 | Diesel (LL) |  | S39 | Mixed organosulfur (LL) |
| S20 | Diesel (SP) |  | S40 | Mixed organosulfur (SP) |

*Each of DBT, BT, 4–MDBT, 4,6–DMDBT, diesel, MgSO_4_, MgSO_4_+DBT and mixed–organosulfur (DBT+4–MDBT+4,6–DMDBT+BT) were used as a sulfur source with glucose as a carbon source. Ethanol and 2–HBP were used as a carbon source with MgSO_4_ as a sulfur source. **EL:** early–log, **ML:** mid–log, **LL:** late–log and **SP:** stationary phase.
